# IgG Subclass Determines Suppression Versus Enhancement of Humoral Alloimmunity to Kell RBC Antigens in Mice

**DOI:** 10.3389/fimmu.2020.01516

**Published:** 2020-07-16

**Authors:** Paurvi Shinde, Heather L. Howie, Tamara C. Stegmann, Ariel M. Hay, Hayley R. Waterman, Zoltan Szittner, Arthur E. H. Bentlage, Linda Kapp, Suzanne N. Lissenberg-Thunnissen, Gillian Dekkers, Richard B. M. Schasfoort, Sarah J. Ratcliffe, Mark E. Smolkin, Gestur Vidarsson, C. Ellen van der Schoot, Krystalyn E. Hudson, James C. Zimring

**Affiliations:** ^1^Bloodworks Northwest Research Institute, Seattle, WA, United States; ^2^Department of Pathology, Carter Immunology Center, University of Virginia School of Medicine, Charlottesville, VA, United States; ^3^Sanquin Research and Landsteiner Laboratory, Department of Experimental Immunohematology, Academic Medical Center, University of Amsterdam, Amsterdam, Netherlands; ^4^Medical Cell Biophysics Group, MIRA Institute, University of Twente, Enschede, Netherlands; ^5^Department of Pathology and Cell Biology, Columbia University Irving Medical Center, New York, NY, United States

**Keywords:** antibody, immune regulation, IgG subclass, red blood cell, alloimmunity

## Abstract

It has long been appreciated that immunoglobulins are not just the effector endpoint of humoral immunity, but rather have a complex role in regulating antibody responses themselves. Donor derived anti-RhD IgG has been used for over 50 years as an immunoprophylactic to prevent maternal alloimmunization to RhD. Although anti-RhD has dramatically decreased rates of hemolytic disease of the fetus and newborn (for the RhD alloantigen), anti-RhD also fails in some cases, and can even paradoxically enhance immune responses in some circumstances. Attempts to generate a monoclonal anti-RhD have largely failed, with some monoclonals suppressing less than donor derived anti-RhD and others enhancing immunity. These difficulties likely result, in part, because the mechanism of anti-RhD remains unclear. However, substantial evidence exists to reject the common explanations of simple clearance of RhD + RBCs or masking of antigen. Donor derived anti-RhD is a mixture of 4 different IgG subtypes. To the best of our knowledge an analysis of the role different IgG subtypes play in immunoregulation has not been carried out; and, only IgG1 and IgG3 have been tested as monoclonals. Multiple attempts to elicit alloimmune responses to human RhD epitopes in mice have failed. To circumvent this limitation, we utilize a tractable animal model of RBC alloimmunization using the human Kell glycoprotein as an antigen to test the effect of IgG subtype on immunoregulation by antibodies to RBC alloantigens. We report that the ability of an anti-RBC IgG to enhance, suppress (at the level of IgM responses), or have no effect is a function of the IgG subclass in this model system.

## Introduction

The use of passively transferred antibodies to RhD (anti-D) as an immunoprophylaxis to prevent maternal alloimmunization represents a highly successful therapeutic intervention to avoid hemolytic disease of the fetus and newborn (at least with regards to RhD). Moreover, use of anti-D is one of only a very few examples of successful immunomodulation preventing reactivity to a specific antigen without inducing general immunosuppression. However, despite its widespread success, the mechanisms by which anti-D prevents alloimmunization to RhD remains obscure. Although a number of popular theories have been put forward, there is substantial evidence to reject both of the most common explanations, that immunization is prevented by avoiding exposure of the immune system through clearance of RhD+ RBCs or masking of antigen (1).

The lack of mechanistic understanding has precluded explanations for a number of phenomena surrounding the use of anti-RhD. Despite proper use, anti-D fails to protect some pregnant women from alloimmunization (2). Moreover, under certain circumstances, anti-D results in enhancement, rather than suppression, of alloimmunization to RhD (3). Finally, attempts to generate a monoclonal anti-D have largely failed, resulting in either decreased efficacy compared to donor derived anti-D, or in some cases, monoclonal anti-D has shown the same paradoxical enhancement of alloimmunization seen with certain preparations of donor derived anti-D (4, 5); although a recent report indicates great progress in this area (6). Why anti-D suppresses alloimmunization in some cases and enhances alloimmunization in others remains an unsolved question.

Humans express 4 different IgG subclasses, each with different effector functions regarding ligation of Fc gamma receptors (FcγRs), fixation of complement, and integration with different biological systems (7). Polyclonal donor derived anti-D is a mixture of all 4 IgG subclasses– each of which may have different functional effects. Only IgG1 and IgG3 have been tested as therapeutic monoclonal anti-D; and, to the best of our knowledge, the possibility that IgG subclass may be an independent variable affecting immunoregulatory effects of anti-D has not been assessed.

Like humans, mice express 4 different IgG subclasses, which differ in orthology but are analogous by function. Herein, we used a mouse model to test the hypothesis that IgG subclass of anti-RBC antibodies affects immunoregulatory function. Systems in which mice make a humoral response to human RhD have remained elusive. As such, we utilized a murine system of humoral immunization to a human blood group antigen (K1 of the Kell system) in combination with a panel of anti-K1 IgG switch variants (i.e., antibodies with identical antigen binding domains but of different IgG subclasses). This model is not intended to exactly represent RhD; rather it serves to test how IgG subclass affects alloimmunity to RBC alloantigens in an analogous system. We report that antibodies with the same antigen binding domain have different immunoregulatory effects based upon IgG subclass.

## Materials and Methods

### Mice

Wild-type (WT) C57BL/6J (B6), Fc-γ-chain^−/−^ (stock# 002847) and UbiC-GFP transgenic (stock #004353) were purchased from the Jackson laboratory (Bar Harbor, ME). K1 and K2 transgenic mice were generated as previously described (8, 9). K1 mice were crossed with UbiC-GFP to allow *in vivo* monitoring by flow cytometry without staining (K1.GFP). All K1 RBCs transfused in this study were from K1.GFP mice; but are simply referred to as K1 in this paper for simplicity of nomenclature. All of these mice were housed and/or bred in Bloodworks Northwest Research Institute vivarium (Seattle, Washington) and all procedures were performed according to approved IACUC protocols.

### Monoclonal Antibodies and Passive Immunization

PUMA1 and PUMA 6 and their switch variants were isolated, expressed, and purified to homogeneity as previously described (10, 11). B6 mice were passively immunized by tail vein injection with 0.25μg of PUMA1 IgG1, IgG2a, IgG2b, IgG2c, or IgG3 in a total volume of 250 μL of PBS, 2 h before the transfusion.

### Transfusion of RBCs and Monitoring RBC Circulation

K1 or B6 RBCs were collected as previously described (12). Prior to transfusion B6 RBCs were labeled with 1,1′-dioctadecyl-3,3,3′3′-tetramethylindocarbocyanine perchlorate (CellTracker™ CM-DiI Dye, Thermo Fisher Scientific) as previously described (12). Fifty microliter each of K1 and DiI labeled B6 RBCs were mixed at 1:1 ratio and the recipient B6 mice were transfused with 100 μL packed RBCs diluted in total volume of 500 μL PBS (20% hematocrit) via tail vein injection. K1 and B6 RBCs were enumerated in peripheral blood and K1 survival was calculated as a ratio of K1:B6 RBCs, as described (12) Because this procedure normalizes the survival of antigen positive RBCs as a function of wild-type (B6) RBCs injected as a mixture, it removes issues surrounding variability of injection, blood volume, or phlebotomy (12).

### Assaying Humoral Immune Responses

Sera were incubated with K1, K2, or B6 RBCs followed by APC conjugated anti-mouse IgM or IgG secondary antibodies purchased from Southern Biotechnology (Homewood, AL).

MFIs were obtained by subtracting MFI of B6 targets from K1 targets for each sample.

### Determination of Affinity of IgG Opsonized RBCs to Fc Receptors

Cellular SPR (cSPRi) measuring binding avidities of IgG-opsonized RBCs with FcγRs spotted on a streptavidin-sensor were carried out on an IBIS MX96 as previously described (13). Whereas traditional SPR methods utilize monomeric proteins binding to a solid matrix coated with their target; cSPR tests how the multiavid nature of proteins binding to a cell surface interacts with the solid matrix. This condition is more representative of the nature of the biochemical interaction that takes place in in the context of antibodies bound to an RBC; as such, the cSPR method provides more relevant information than SPR on monomeric immunoglobulin.

### Statistical Analysis

Each outcome was modeled via a generalized estimating equation (GEE), mixed effects or tobit model, as appropriate. Log-transformations were applied as necessary for normality assumptions. Models were adjusted for potential experimental effects and repeated observations within a mouse. Estimated marginal means were used for pairwise comparisons with PBS, with Dunnett adjustments for multiple comparisons.

## Results

### IgG Subclass of Anti-RBC Immunoglobulin Regulates Suppression vs. Enhancement

**Figure 1:**
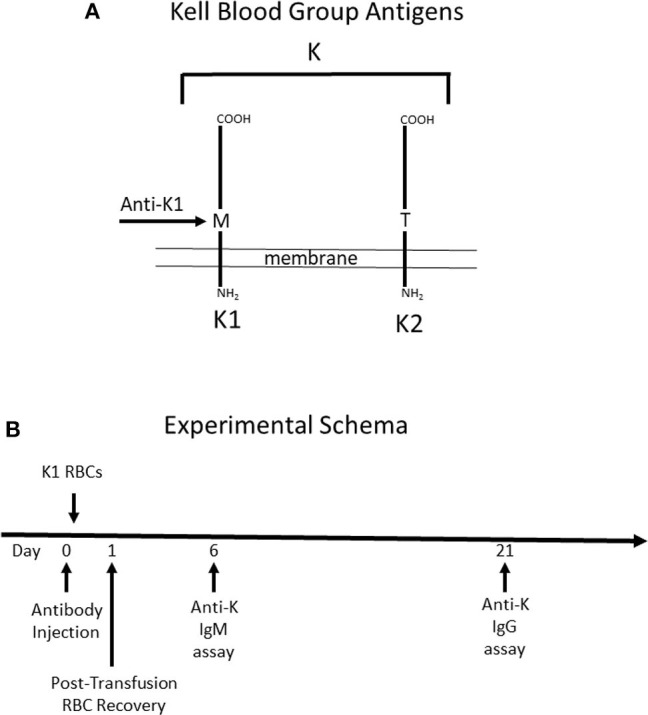
Schematic Description of Mice used and Experimental Design. **(A)** Mice expressing the human Kell glycoprotein as a transgene were utilized for this study. Two different mice were used. The first mouse (K1) expresses the K1 variant of the Kell glycoprotein and the (K2) mouse expresses the K2 variant—defined as a methionine vs. threonine at position 193, respectively. Anti-K1 (PUMA1) binds to the K1 but not the K2 variant. **(B)** The experimental design consisted of injecting antibody (or control PBS) at time point zero, followed by K1 RBCs 2 h later. When transfusing into wild-type recipients, the majority of the immune response is against antigens on the Kell glycoprotein other than K1 and K2, as such the immune response is referred to as anti-K. Anti-K IgM and IgG were measured at 6 and 21 days post-transfusion, respectively.

To test the role that IgG subclass plays in regulating humoral immunity to RBC antigens, we focused on the Kell blood group system (Figure 1A). The Kell glycoprotein is a type II transmembrane protein that carries at least 35 alloantigens defined by single amino acid variations between humans (14). K1 and K2 are the most clinically important in the Kell system, and are antithetical antigens defined by a methionine (K1) or threonine (K2) at position 193. IgG switch variants *(i.e., same antigen binding domain but different IgG subclasses)* of a monoclonal anti-K1 (PUMA1) were expressed recombinantly and purified to homogeneity (10). Each IgG subclass (IgG1, IgG2a, IgG2b, IgG2c, and IgG3) was individually injected into mice on day 0. Two hours later, mice were transfused with RBCs from transgenic mice expressing K1 (9). RBC clearance was determined at 24 h by enumerating circulating K1 RBCs in peripheral blood by flow cytometry. Serum was then collected and tested for alloantibodies to K1 RBCs; IgM was assayed at day 6 and IgG was assayed at day 21.

It is important to note that while mice express an ortholog of human Kell, 25% of the amino acid residues differ between species (15). Thus, wild-type mice have the capacity to respond to multiple epitopes and/or antigens other than K1 that are carried by the Kell glycoprotein. Antibody responses by treated mice will be referred to as anti-K, to reflect a general response to the entire K glycoprotein. Injected anti-K1 will be referred to as mAb anti-K1.

Day 21 was chosen as the time point to analyze serum for anti-K IgG, out of concern that signal from injected mAb anti-K1 may be mistaken for anti-K made by the recipient immune response. This concern was also addressed by including control groups that were injected with mAb anti-K1 but received no transfusion; baseline signal was determined from these control groups at the same time points as experimental groups.

**Figure 2:**
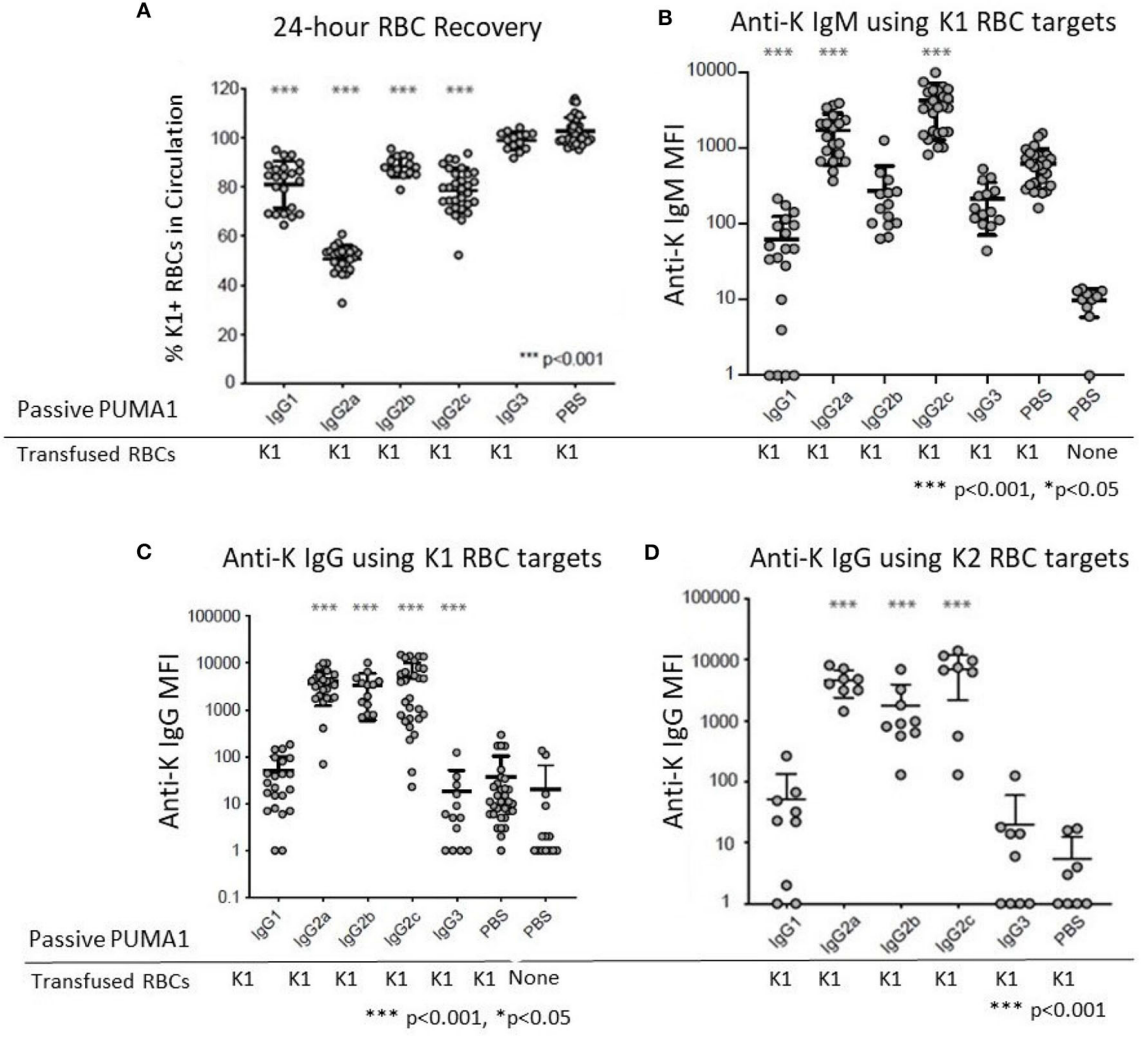
Differential Effect of IgG Subclass on Immunoregulatory Properties of anti-RBC Antibodies. **(A)** 24 h recoveries of K1 RBCs were assessed following transfusion in response to the indicated antibodies or control PBS **(B)** Anti-K IgM was measured at 6 days post-transfusion. **(C)** Anti-K IgG was measured at 21 days post-transfusion. **(D)** The same 21-day serum tested in panel C were analyzed for IgG using K2 RBCs as targets rather than K1, in order to avoid interference from the injected mAb anti-K1. These data are pooled from 3 to 10 experiments with *n* = 9–23 mice per group. Statistically significant differences from PBS-treated mice are shown as *p < 0.05 and ***p < 0.001.

Compared to control “compatible” mice injected with PBS, a statistically significant clearance of K1 RBCs was observed with mAb anti-K1 of the IgG1, IgG2a, IgG2b, and IgG2c subclasses (*p* < 0.001 for each subclass) (Figure 2A). The greatest clearance was observed with IgG2a (~50%) and the rank order of clearance was IgG2a>IgG2c>IgG1>IgG2b. No clearance was observed with the IgG3 subclass.

Transfusion of K1 RBCs induced a strong IgM anti-K response at 6 days post-transfusion. In the absence of any injected mAb anti-K1 (PBS group) the IgM signal was 63 times stronger (Figure 1B) than in negative control mice receiving PBS without transfused RBCs. Injection of IgG1 caused significant suppression of the IgM anti-K response (*p* < 0.001). A milder suppression was also observed in response to IgG3 (*p* < 0.05). In contrast, both IgG2a and IgG2c caused substantial enhancement of anti-K IgM (*p* < 0.001). No significant change in anti-K IgM was observed in response to IgG2b injection.

Consistent with previous reports, transfusion of K1 RBCs causes only a very weak IgG response in the absence of adjuvant (8). Low levels of anti-K IgG were detected in mice receiving only K1 RBCs (Figure 2C) compared to mice receiving no RBCs (PBS group in Figure 2C). Similar to the effects upon anti-K IgM, both IgG2a, and IgG2c caused a substantial enhancement of anti-K IgG (*p* < 0.001). Surprisingly, IgG2b also caused enhancement of the same magnitude and significance as IgG2a and IgG2c, despite having no detectable effect on anti-K IgM (see Figure 2B). Neither IgG1 nor IgG3 had any effect upon anti-K IgG. However, as there was no significant baseline IgG response, it is not possible to determine if IgG1 and IgG3 suppressed anti-K IgG.

The observed enhancement was not an artifact of detecting the mAb anti-K1 IgG switch variants that were injected, as 100-fold lower signal was detected in control mice that received mAb anti-K1 but no RBC transfusion (data not shown). More importantly, sera was also analyzed using K2 RBCs as targets (9). K2 RBCs have all epitopes in common with K1 RBCs, except they lack K1 and are thus non-reactive with mAb anti-K1 (10). Accordingly, this assay will detect polyclonal anti-K responses other than anti-K1 and do not detect injected mAb anti-K1. The same results were obtained using K2 RBCs as targets as when K1 RBCs were used (Figure 2D); as such, we interpret the enhanced anti-K to be of recipient origin and reject the interpretation that any of the enhancement seen by injection of mAb anti-K1 IgG2a, IgG2b, or IgG2c was due to detecting the injected mAb anti-K1.

### Both RBC Clearance and Enhancement by IgG2a and IgG2c Requires the Fc Gamma Receptor Common Gamma Chain

**Figure 3:**
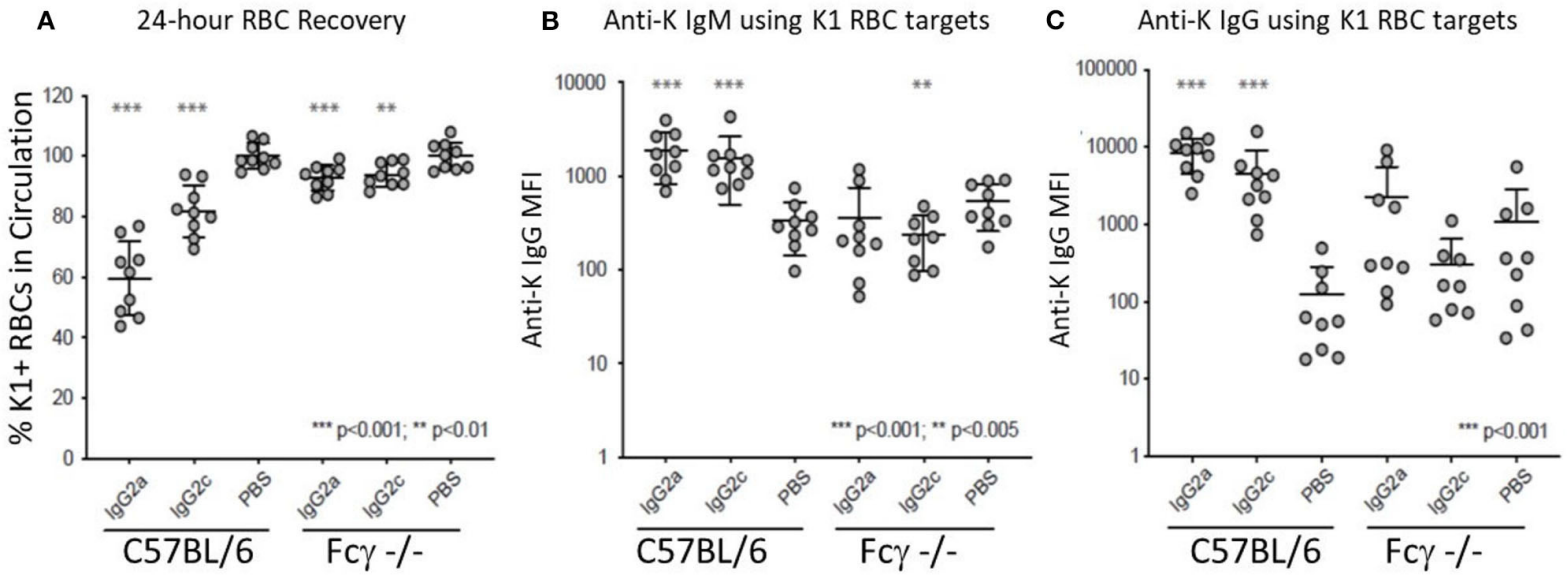
FcγRs are required for IgG2a and IgG2c mediated antibody enhancement. B6 and Fc-γ-chain^−/−^ mice were passively immunized with mAb anti-K1 IgG2a, IgG2c, or PBS followed by transfusion with K1 RBCs. **(A)** 24 h recoveries of K1 RBCs were assessed following transfusion. **(B)** Anti-K IgM was measured on days 6 post-transfusion. **(C)** Anti-K IgG was measured on day 21 post-transfusion. Statistically significant differences from PBS-treated mice are shown as *p < 0.05, **p < 0.01, ***p < 0.005 and ****p < 0.0005.

IgG2a and IgG2c are both known to interact strongly with Fc gamma receptors (FcγR). Mice have four known FcγRs (FcγRI, FcγRII, FcγRIII, and FcγRIV). FcγRI, FcγRIII, and FcγRIV each signal through a common gamma chain and are each considered stimulatory receptors. To test the role of these FcγRs on the observed biology, we used mice with a deletion of the common gamma chain (Fc-γ-chain^−/−^) and compared them to wild-type mice using the same experimental design (see Figure 1B). Clearance of K1 RBCs by IgG2a and IgG2c was greatly decreased in γKO mice compared to B6 controls (Figure 3A). Likewise, enhancement of both IgM (Figure 3B) and IgG (Figure 3C) anti-K by IgG2a and IgG2c was eliminated in γKO mice.

### Affinity of IgG Subclasses for Different FcγRs

Relative affinities of mouse IgG subclasses for different Fc receptors have been described using fluid phase surface plasmon resonance, with the exception of IgG2c, which has not been reported to the best of our knowledge. However, it has been demonstrated that affinity measurements can differ when antibodies are bound to cellular targets as opposed to when they are in a monomeric fluid phase. Moreover, little information exists on the affinity of IgG2c. To investigate affinities of mAb anti-K1 switch variants for FcγRs when they are bound to K1 RBCs, a flow-based method of cellular surface plasmon resonance (cSPR) was used. K1 RBCs were incubated with a titration of each mAb anti-K1 switch variant and then passed over immobilized recombinant FcγRs and affinity was determined as a function of delayed flow. Anti-mIgG reactive with each IgG subclass was used as a positive control whereas BSA (or buffer alone) were used as negative controls.

**Figure 4:**
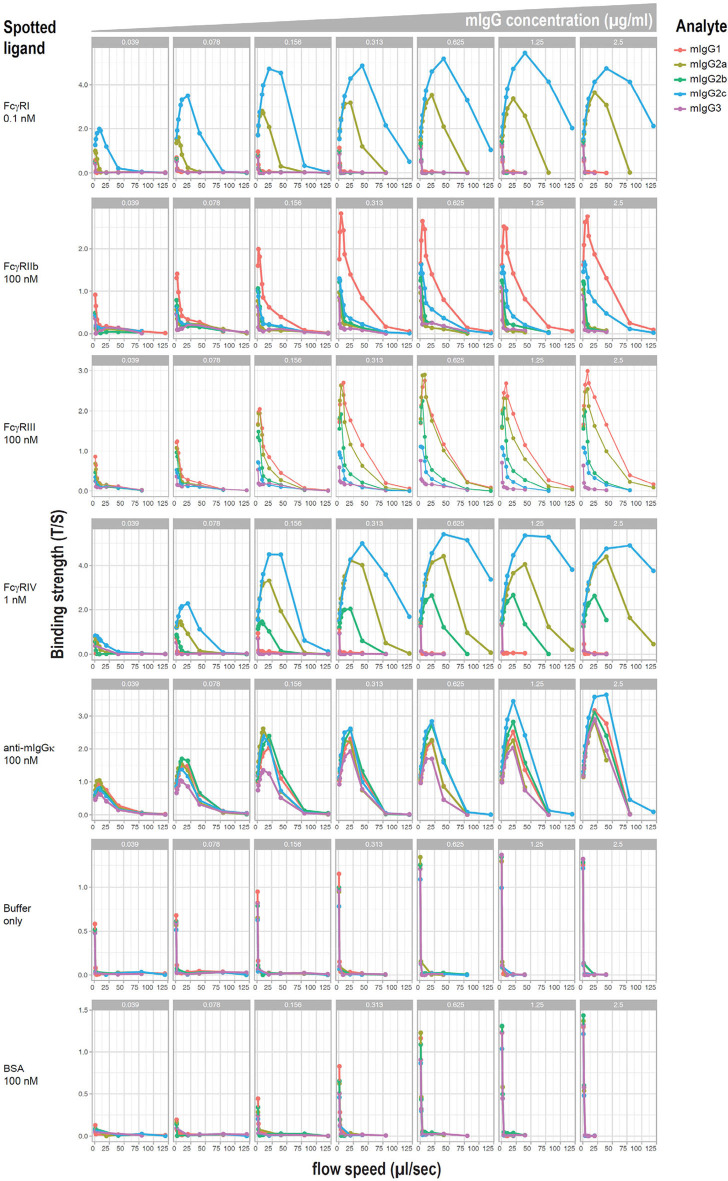
cSPR measurements for IgG subclasses and murine FcγRs. K1 RBCs were saturated with each of the indicated anti-K1 IgG subclasses and passed over solid matrix spotted with the indicated recombinant FcγRs. Anti-mIg and BSA were used as positive and negative controls, respectively.

Representative plots of each switch variant are shown (Figure 4). Focusing on the activating receptors (i.e., FcγRI, FcγRIII, and FcγRIV) IgG2a and IgG2c were the only subclasses that reacted with all 3 activating FcγRs. IgG2c was more reactive with FcγRI and FcγRIV than was IgG2a, whereas IgG2a was more reactive with FcγRIII. IgG2b reacted only with FcγRIV and FcγRIII and had relatively weak binding. IgG1 reacted only with FcγRIII. IgG3 had no detectable reactivity with any FcγR. Focusing on the only known FcγR that is generally considered an inhibitory receptor (FcγRIIb). IgG1 had considerable reactivity and IgG2c had very weak reactivity—no other subclasses showed FcγRIIb reactivity. These data are summarized in Table 1.

**Table 1:** Quantitative representation of affinity of RBC bound IgG subclasses for FcγRs.

	**mIgG1**	**mIgG2a**	**mIgG2b**	**mIgG2c**	**mIgG3**
mFcγRI	–	+	–	++	–
mFcγRIIb	++	–	–	+/–	–
mFcγRIII	++	++	+	+/–	–
mFcγRIV	–	+	+/–	++	

*This table provides a synopsis derived from the data shown in Figure 4*.

## Discussion

The studies presented in this manuscript isolate IgG subclass as an independent variable that can alter the immunoregulatory effect(s) of an antibody upon a primary humoral immune response to an RBC alloantigen. The most important finding from this approach is that IgG subclass can determine if an antibody enhances, suppresses, or has no effect upon a humoral response to an RBC alloantigen. However, there are a number of caveats to our interpretation that IgG subclass is causing the differences. First, we assume that by changing the IgG subclass, through recombinant methods, that this is the only change we have made. It is possible that allosteric effects of subclass alter the affinity of the antibodies or other biochemical/biological properties—and these are not assessed in the current study. Such would still reflect an effect of subclass, but it would be more indirect than a mechanism by which the subclass Fc domain was what altered the biology. It is also important to note that we chose two specific timepoints to measure antibody responses and did not measure this as a continuous variable over time. Thus, while we observed clear differences as a function of IgG subclass, we cannot rule out the possibility that responses were shifted in their kinetics, as opposed to a categorical alteration in response. Nevertheless, the time points we measured are consistent with known kinetics of antibody response to both the K1 alloantigen, as well as other RBC alloantigens that have been assessed in murine systems measured at multiple timepoints over a 28 day period (8, 16).

The ability of some IgG subclasses (i.e., IgG2a, IgG2b, and IgG2c) to enhance alloimmunity to an RBC antigen contradicts the current “dual effect” model, in which IgG against soluble antigens enhances immunity whereas IgG against an RBC alloantigen suppresses (17). The dual effect model has been based upon numerous reports in which IgG can enhance responses to soluble antigens, combined with separate reports in multiple animal models that IgG against RBC antigens is suppressive (18–29) and the well-known suppressive effects of anti-RhD in humans. In particular support of the dual effect model, Enriquez-Rincon and Klaus reported that the same monoclonal antibodies (against a hapten) enhance humoral immunity to a haptenated soluble protein but suppress responses to haptenated RBCs (30). However, the dual effect model has also been contradicted by data in rabbits and humans that IgG against RBCs can enhance humoral immunity (3–5, 31). However, none of these reports considered how IgG subclasses may affect responses. The current report may serve to help refine the dual effect model, by demonstrating that “IgG” cannot be treated as a single entity, and that the IgG subclass affects the immunoregulatory result.

The observed ability of IgG2a and IgG2c to enhance alloimmunity to RBC alloantigens in mice is not particular to the K1 system or the PUMA1 antibody; we recently reported a similar effect using a different RBC alloantigen (HOD RBCs) and a distinct panel of switch variants from a monoclonal antibody recognizing a Duffy epitope on the HOD RBCs (PUMA6) (11). In this case, IgG2a, and IgG2c (but not IgG2b) enhanced IgM and IgG responses to HOD. Thus, based upon the K1/PUMA1 and HOD/PUMA6 systems, one might conclude that the enhancement of anti-RBC by IgG2a and IgG2c is a generalizable principle, at least in mice. However, Yu et al. (29) carried out an analysis of a panel of different monoclonal antibodies to a model RBC alloantigen (*i.e.*, the HOD antigen). Of the different monoclonals tested, 4 were IgG1 and 1 was IgG2a and 1 was IgG2b—all 6 of the tested antibodies resulted in suppression. No IgG2c or IgG3 were tested in this study.

Why IgG2a and IgG2b enhanced in the current (and recent) reports (11) but suppressed in the report by Yu et al. (29) is unclear. However, it is worth noting that each of the antibodies used by Yu et al. (29) had different antigen binding domains, different affinities, and bound to different epitopes; as such, it is not possible to isolate IgG subclass as an independent variable. It is also important to note that the current studies did not include a control IgG of each subclass but against a third party antigen not in the system so as to rule out non-specific effects. However, the same antibody used in these studies (PUMA1) had no effect in the IgG2c form when used in the presence of a non-K1 RBC alloantigen in a separate study (11). Future studies will be required to address if and how affinity, epitope target, and other possible factors may also regulate suppressing vs. enhancing effects separate from IgG subclass.

Several mechanistic inferences can be drawn from the current data. First, similar to other studies in mice and in humans, clearance of RBCs is not alone sufficient to explain immunoregulatory effects—and may play no mechanistic role at all. K1 RBCs were cleared by IgG1 and IgG2c to a similar extent, but the former suppressed whereas the latter enhanced. With regards to mechanisms of enhancement, we focused on IgG2a and IgG2c. Both IgG subclasses caused clearance, both caused enhancement, and deletion of FcγRs eliminated both clearance and enhancement. However, IgG2a caused significantly more clearance but less enhancement than IgG2c. Thus, clearance does not correlate with either extent of enhancement or the issue of enhancement vs. suppression.

The detailed analysis of IgG2c in the current case is of significance. Whereas, IgG2a is expressed by most commonly used inbred strains of mice, some strains express IgG2c instead of IgG2a (most notably C57BL/6), presumably as an allotypic variation of the same genetic locus (32). Monoclonal antibodies of the IgG2a subclass are commonly used in mice on a C57BL/6 background: most antibody tools are not available in the IgG2c subclass. However, it has been reported that when an IgG type that expresses non-self sequence is used to suppress humoral responses to RBC alloantigens, then the immune response can shift away from the RBC and to foreign epitopes in the antibody used (21). Thus, the inclusion of IgG2c in the current studies is of considerable importance, as IgG2c is immunological “self” to a C57BL/6 mouse but the constant region of IgG2a has genetic variation leading to alloantigens. IgG2c is far less studied than is IgG2a and thus limited information is available concerning its affinity for different FcγRs. However, cSPR studies performed in this paper provided a relative affinity profile for each IgG subclass (including IgG2c) and in the context of antibodies bound to an RBC.

FcγRs are required for both clearance and enhancement, as both were lost in the Fc-γ-chain^−/−^ mouse. Although the functional effects of individual FcγRs were not evaluated, specific hypotheses can be derived from the cSPR data. IgG2a, IgG2b, and IgG2c were the only subclasses that enhanced IgG responses and they are the only subclasses that bound to FcγRIV. Thus, FcγRIV emerges as a prime candidate. These subclasses also bound to FcγRI and FcγRIII; however, we postulate that these receptors are less involved. First, FcγRI is a high affinity receptor that tends to bind monomeric IgG, and as such, it is often discounted as being important in responses to antibody opsonized RBCs as FcγRI should be saturated by monomeric IgG. Second, FcγRIII was bound by IgG1, which did not enhance. However, this interpretation is complicated when considering that IgG1 bound to FcγRIIb, which is an inhibitory receptor. Thus, it is not possible from the current data to interpret if the inhibitory effects of IgG1 binding FcγRIIb may have offset any activating signal from FcγRIII.

The issue of what role inhibition by FcγRIIb may be playing is an important consideration. It is possible that IgG1 suppresses because it is has the highest affinity for FcγRIIb and also does not interact with FcγRIV. Moreover, the binding of FcγRIIb by IgG2c but not IgG2a may explain why IgG2c has lower enhancing activity than IgG2a. FcγRIIb has been discounted as important in suppression of anti-RBC responses because deletion of FcγRIIb failed to prevent suppression in at least one system (19). However, the role that FcγRIIb is playing in the current setting, if any, is not yet clear.

It is curious that IgG2b enhanced IgG without an observed effect upon IgM, likely indicating a different mechanism of enhancement by IgG2b than by IgG2a or IgG2c. It is also notable that IgG2b had significantly lower clearance than either IgG2a or IgG2c. Moreover, IgG2a and IgG2c bound to FcγRI whereas IgG2b does not. However, other than these correlations, the mechanistic underpinnings that would lead to the serological differences remain unclear and will require further investigation. However, the observations do support the notion that more than one mechanism of enhancement is likely at play amongst different IgG subclasses.

In light of the current findings that IgG subclass can determine suppression vs. enhancement, at least in mice, the IgG subclass composition of therapeutic donor derived human anti-D becomes a relevant question. At least one manufacturer (CSL Behring UK Limited) listed the IgG composition of its product (Rhophylac) as IgG1 (84.1%), IgG2 (7.6%), IgG3 (8.1%), IgG4 (1.0%) (33). It is unclear from these publicly available data whether this represent total IgG subclass composition as opposed to IgGs with an anti-RhD specificity. However, these numbers are consistent with published literature on IgG subclasses of anti-RhD found in alloimmunized humans. Although It has been reported by some that only IgG1 and IgG3 are present in anti-RhD induced by pregnancy or transfusion (34, 35), others have clearly found IgG2 at a similar frequency as IgG3. This discrepancy can be explained in that there is considerable variability amongst individuals. For example, a single IgG subclass was present in 64.5% of individuals, including some individuals with isolated IgG2 or IgG4 (36) and other studies have detected IgG2 and IgG4 in alloimmunized individuals (37–39). Nevertheless, as therapeutic preparation of anti-RhD are pooled from multiple donors, it seems reasonable to infer that the general composition is a predominance of IgG1, followed by lesser (but roughly equal IgG2 and IgG3) and scant amounts of IgG4.

The issue of what is injected vs. what is bioavailable is of considerable importance. To the best of our knowledge, how the different IgG subclasses enter circulation after intramuscular injection in humans has not been studied. However, pharmacokinetics studies in mice indicate that the ability of IgG to bind the FcRn affect how well it gets into circulation after subcutaneous injection (40). Since each of the human IgG subclasses binds well to FcRn (7), one might infer that they will equally enter circulation—although this remains to be tested empirically. However, equally important is that IgG1, IgG2, and IgG4 have a 21 day half-life, whereas the half-life of IgG3 is only 7 days (although this can vary based upon isoallotype) (7). Due to this half-life issue, IgG1 may remain the major component, but IgG2 (and whatever IgG4 may be present) may then exceed IgG3.

Numerous monoclonal anti-D have been tested in humans, and historically none have suppressed as well as donor derived anti-D, whereas some have enhanced alloimmunization to RhD. To the best of our knowledge, only IgG1, and IgG3 have been tested. This has been due to several factors. First, this was due to the focus on isolating IgG subclasses that cause RBC clearance due to the prevailing theory at the time that indicated RBC clearance to be an indicator of immunoprophylaxis—a theory that is currently quite controversial (1). Second, it seems clear that once a mother is immunized, IgG1, and IgG3 are the predominant forms that cause HDFN (41, 42). However, induction of HDFN and suppression of afferent immunity are different biological effects, and it is unclear why they would involve the same mechanisms, especially if clearance of RBCs is not responsible in suppression. Finally, this may have been guided by early reports that donor derived anti-D contained only IgG1 and IgG3 (34); a finding that (as above) does not hold after study of a broader population of humans (36–39).

The data presented herein raise the question as to whether IgG subclass of anti-D will affect immune regulation of anti-D in humans. Given that IgG2 and IgG3 are present in roughly equal amounts in donor derived anti-D, and IgG2 has a much longer half-life than IgG3, then it is reasonable to predict that IgG1 and then IgG2 are the most prevalent IgG subclasses in donor derived anti-D; yet, only IgG1, and IgG3 (and not IgG2) have been tested as monoclonals, driven by a thought process that is logical, but likely based upon a faulty premise (e.g., that clearance is the mechanism of suppression so subclasses that best promote clearance should be used). We suggest that IgG2, and even IgG4, may be contributing significantly to the effects of anti-D and that exclusion of their study may contribute to the failure of efficacy of monoclonal anti-D. Future research in humans should test this hypothesis.

## Data Availability Statement

The raw data supporting the conclusions of this article will be made available by the authors, without undue reservation.

## Ethics Statement

The animal study was reviewed and approved by BloodworksNW Research Institute IACUC.

## Author Contributions

The experiments were designed by PS, TS, GV, CS, KH, and JZ. Experiments were carried out by PS, HH, TS, AH, HW, ZS, AB, LK, SL-T, GD, and RS. SR and MS carried out statistical analysis. All authors were involved in data interpretation and all authors contributed to the writing of the manuscript. All authors contributed to the article and approved the submitted version.

## Conflict of Interest

The authors declare that the research was conducted in the absence of any commercial or financial relationships that could be construed as a potential conflict of interest.
